# Scurvy: A Diagnosis Not to Be Missed

**DOI:** 10.7759/cureus.33050

**Published:** 2022-12-28

**Authors:** Yazmeen Tembunde, Shealinna Ge, Kathryn Turney, Marcia Driscoll

**Affiliations:** 1 Dermatology, University of Maryland School of Medicine, Baltimore, USA

**Keywords:** petechial rash, malnutrition, ascorbic acid, vitamin c, scurvy

## Abstract

Vitamin C deficiency, one of the oldest-known nutritional disorders, is now uncommon in high-income countries. Recently, however, there has been an increase in cases of vitamin C deficiency, also known as scurvy. We report three adult patients with histories of homelessness, food insecurity, and poor nutrition, making them particularly vulnerable to restrictive diets and at increased risk for scurvy. After proper diagnosis and treatment, favorable outcomes can be rapidly obtained. This case series emphasizes the importance of keeping a broad differential diagnosis and inquiring about nutritional history in patients presenting with purpura, gingival bleeding, and body hair changes.

## Introduction

Once a severe health problem decimated explorers of the 15th and 16th centuries, hypovitaminosis C, colloquially known as scurvy, is now uncommon in high-income countries [1]. However, there has been an unexpected increase in the incidences of hypovitaminosis C in recent years. Vitamin C (ascorbic acid) plays a vital role in human physiology; it is involved in collagen synthesis, iron absorption, antioxidant processes, and other biochemical pathways. Vitamin C deficiency results in defective collagen synthesis, causing blood vessel fragility and the critical findings of petechiae, ecchymosis, bleeding gums, and hemarthrosis [2,3]. We can obtain vitamin C through fresh fruit and vegetables in our diet, and inadequate fruit or vegetable intake can lead to vitamin C deficiency and, subsequently, scurvy [2]. We present three adult cases of scurvy that occurred within the past year. In one of these cases, many evaluations with laboratory testing and imaging were made before the dermatologic consult.

## Case presentation

Case 1

**Figure 1:**
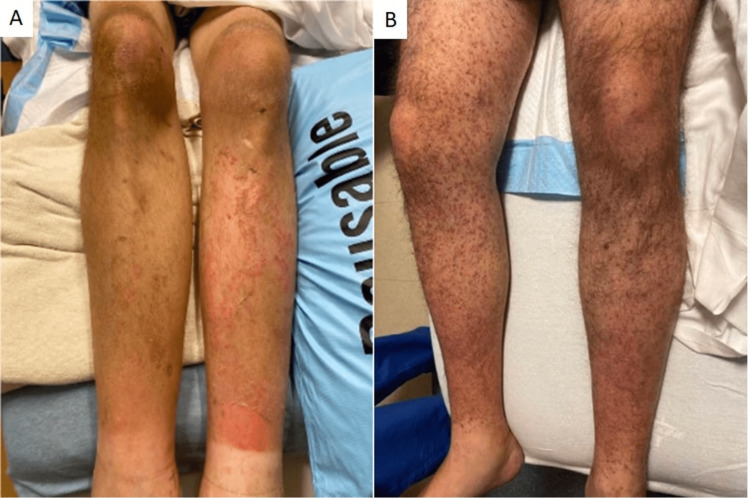
A) patient 1 with non-blanching perifollicular purpura on bilateral lower extremities. B) patient 2 with folliculocentric hyperkeratotic petechial papules and scattered corkscrew hairs

A 35-year-old man with a history of homelessness for the past several years with minimal access to food presented to the emergency department (ED) with diffuse body pains and lower extremity purpura. On admission, he was severely malnourished (body mass index 13.29kg/m²) with generalized muscle wasting. Examination of the bilateral lower extremities showed purpura in a perifollicular distribution (Figure 1A). Laboratory results showed normocytic anemia (Hemoglobin 9.8g/dL), normal platelet count and coagulation studies, and absence of cryoglobulins. Plasma ascorbic acid concentration was low at 0.1 mg/dL [4]. Within 2 weeks of initiating 1000mg oral daily vitamin C supplementation, the patient's vitamin C concentration normalized, and the lower extremity purpura resolved.

Case 2

**Figure 2:**
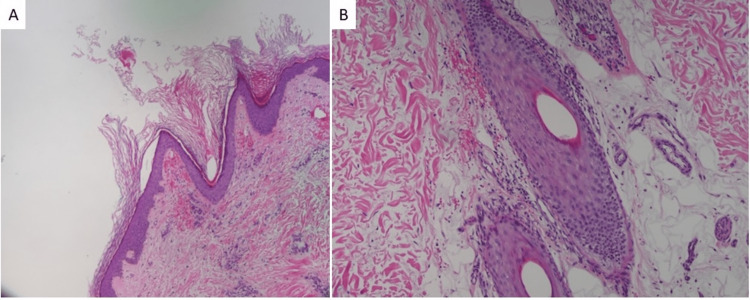
Punch biopsy showing stratum corneum hyperkeratosis with perifollicular erythrocyte extravasation and a scant superficial perivascular mononuclear infiltrate (40x)

A 30-year-old man presented to the ED with a bilateral lower extremity rash that began 1 month prior. The rash started with numerous small red bumps on the lower legs that spread to the thighs. Two weeks later, the patient noticed a rash on his bilateral forearms. He reported lower back pain, right knee pain, and bruising around the time of the rash presentation, with painful bleeding gums and easy bruising more recently. He reported that his diet included limited fruits and vegetables. X-rays of the lumbar spine and right knee showed no abnormalities. The appearance of the rash was unchanged after completing a 2-week course of doxycycline and prednisone prescribed at the ED. The patient was subsequently seen by dermatology and noted to have scattered non-blanching folliculocentric hyperkeratotic red papules and scattered "corkscrew" hairs (Figure 1B). A skin biopsy of his right lower leg showed a hyperkeratotic stratum corneum with perifollicular and peribulbar extravasation of erythrocytes, and a scant nonspecific superficial perivascular mononuclear infiltrate (Figures 2A, 2B). His ascorbic acid concentration (<0.1 mg/dL) and hemoglobin (8.0 g/dL) were low. He was promptly started on 1000 mg of oral vitamin C supplementation daily but was lost to follow-up.

Case 3

**Figure 3:**
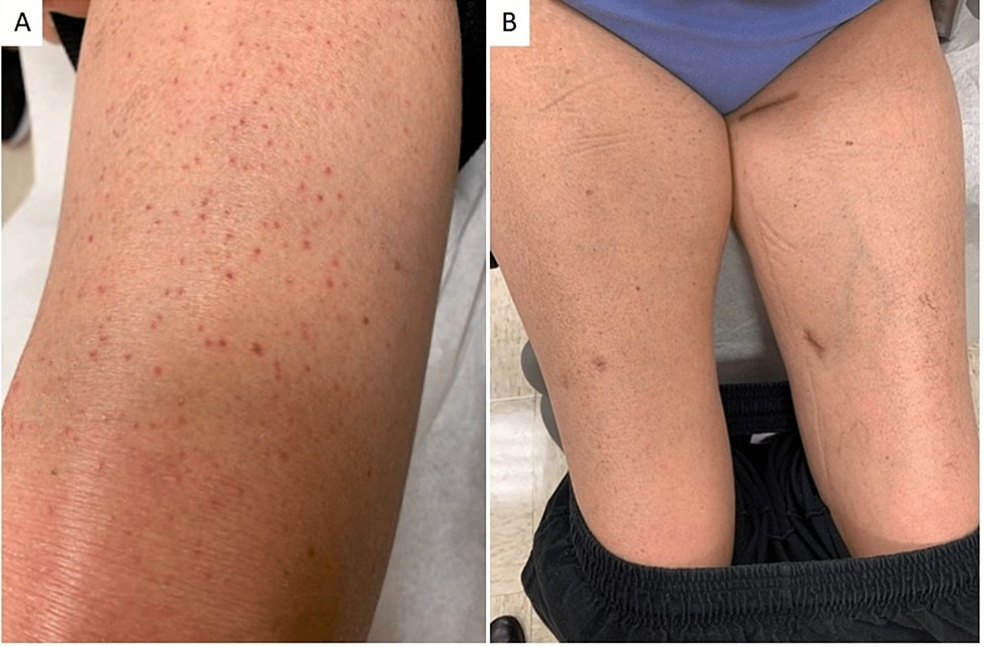
A) patient 3 at initial presentation with non-blanching folliculocentric hyperkeratotic petechial papules on bilateral lower extremities and B) with the resolution of cutaneous findings after 2 months of vitamin C supplementation

A 52-year-old woman presented to the dermatology clinic for evaluation of bilateral lower extremity purpura, joint pain, decreased mobility, and skin fragility on the upper arms. The patient indicated that her diet had been limited due to dental pain. Prior rheumatology and infectious workup were largely unremarkable. The dermatologic exam showed non-blanching folliculocentric petechial lesions of both legs up to thighs and purpuric macules that coalesced into erythema on both feet (Figure 3A). A biopsy of her left lower leg revealed pigmented purpura without evidence of vasculitis or eosinophilic fasciitis. Laboratory testing showed mild normocytic anemia (11.4g/dL), normal platelet count and coagulation studies, and the absence of cryoglobulins. A deficient serum ascorbic acid concentration was noted (<0.1mg/dL). The patient was treated with 1000mg of oral vitamin C supplementation daily. At a follow-up visit 2 months later, her serum ascorbic acid level was normal. Her lower extremity findings resolved (Figure 3B), and she reported improving her joint pain and mobility.

## Discussion

Scurvy is one of the oldest-known nutritional disorders. It became a severe problem in the 15th century, causing significant morbidity and mortality among explorers on long sea trips with limited access to vitamin C-rich foods, such as citrus fruit or vegetables [1,5]. The prevalence of vitamin C deficiency in the United States is 7.1% [6]. Populations at risk include low incomes, food insecurity, poor nutrition, alcohol use disorder, gastrointestinal disorders and malabsorption, and eating disorders [5]. Studies estimate that among people with low incomes, there is a 40% prevalence of vitamin C deficiency [7]. Our patients' histories of homelessness, food insecurity, and poor nutrition made them particularly vulnerable to having restrictive diets and to a heightened risk for scurvy. In one case, the primary team treating the patient did not consider the diagnosis of scurvy, and extensive evaluations were performed before a dermatology consultation was made.

Signs and symptoms of scurvy usually develop after 1-3 months of insufficient vitamin C. Vitamin C plays an integral role in several biochemical pathways, such as collagen biosynthesis and iron absorption [3]. Mature collagen is composed of three polypeptide molecules that form a triple helix. Vitamin C is needed as a cofactor in the hydroxylation of lysine and proline residues on the polypeptides to allow for the formation of the triple helix structure [8]. If this reaction does not occur, the polypeptides are unstable and unable to form rigid, triple helices. This collagen abnormality leads to blood vessel fragility and poor wound healing [3]. Defective collagen synthesis and poor iron absorption are responsible for scurvy's many clinical signs and symptoms, such as easy bruising, petechiae, bleeding gums, myalgia, anemia, hemarthrosis, perifollicular hemorrhages, and corkscrew hairs [9,10]. Extracutaneous hemorrhage may also occur in muscles, bones, eyes, heart, and the nervous system [11-14], resulting in hematomas, subperiosteal bleeding, fractures due to osteopenia, loosening and subsequent loss of teeth, conjunctival varicosities, retrobulbar hemorrhages, hemopericardium, cardiac tamponade, and neuropathy due to hemorrhage into nerve sheaths [12,15-17].

Given that vitamin C and folate are often found in the same foods and that vitamin C also promotes iron absorption, patients who are deficient in vitamin C are often iron and folate-deficient [18]. Anemia is seen in 75% of patients with scurvy, making it the most common laboratory finding in scurvy [2,17]. All three of our patients were anemic. Anemia in scurvy may be due to hemorrhage into tissues, hemolysis, or coexisting iron and folate deficiencies [18].

The diagnosis of scurvy can be confirmed by plasma or serum ascorbic acid level testing [12]. Ascorbic acid levels less than 0.2mg/dL (10 μmol/L) are consistent with severe deficiency [4]. Skin biopsies are often done to aid in diagnosis. Biopsy specimens of skin lesions often demonstrate follicular hyperkeratosis, perifollicular hemorrhage, a proliferation of blood vessels, and coiled hair follicles [12]. The diagnosis of scurvy is frequently delayed or overlooked because of its rarity and can lead to unnecessary exhaustive workups [5]. The differential diagnosis can be broad, encompassing other causes of hemorrhage, purpura, and joint effusion. This includes coagulation disorders, vasculitis, idiopathic thrombocytopenic purpura, rheumatoid arthritis, and septic arthritis [2].

Although its complications can be severe, the treatment of scurvy is simple and safe with daily supplementation of 300-1000mg of vitamin C [2] with a resolution of symptoms within days to weeks of the start of treatment [2,12]. The patients in this case series did not exhibit any side effects with the 1000mg dose.

## Conclusions

Despite being considered a disease of the past, scurvy still occurs in the modern era and can be quickly and safely treated. We present 3 cases of scurvy seen within 11 months at a single academic center, emphasizing the importance of keeping a broad differential diagnosis. These cases remind clinicians to inquire about nutritional history in patients presenting with purpura, gingival bleeding, and body hair changes, mainly if they belong to an at-risk population.

## References

[1] Magiorkinis E, Beloukas A, Diamantis A (2011). Scurvy: past, present and future. Eur J Intern Med.

[2] Montalto M, Porceddu E, Pero E (2021). Scurvy: a disease not to be forgotten. Nutr Clin Pract.

[3] Léger D (2008). Scurvy: reemergence of nutritional deficiencies. Can Fam Physician.

[4] Levavasseur M, Becquart C, Pape E, Pigeyre M, Rousseaux J, Staumont-Sallé D, Delaporte E (2015). Severe scurvy: an underestimated disease. Eur J Clin Nutr.

[5] Khalife R, Grieco A, Khamisa K, Tinmouh A, McCudden C, Saidenberg E (2019). Scurvy, an old story in a new time: the hematologist's experience. Blood Cells Mol Dis.

[6] Schleicher RL, Carroll MD, Ford ES, Lacher DA (2009). Serum vitamin C and the prevalence of vitamin C deficiency in the United States: 2003-2004 National Health and Nutrition Examination Survey (NHANES). Am J Clin Nutr.

[7] Mosdøl A, Erens B, Brunner EJ (2008). Estimated prevalence and predictors of vitamin C deficiency within UK's low-income population. J Public Health (Oxf).

[8] Hall SL, Greendale GA (1998). The relation of dietary vitamin C intake to bone mineral density: results from the PEPI study. Calcif Tissue Int.

[9] Smith A, Di Primio G, Humphrey-Murto S (2011). Scurvy in the developed world. CMAJ.

[10] Velandia B, Centor RM, McConnell V, Shah M (2008). Scurvy is still present in developed countries. J Gen Intern Med.

[11] Pangan AL, Robinson D (2001). Hemarthrosis as initial presentation of scurvy. J Rheumatol.

[12] Hirschmann JV, Raugi GJ (1999416). Adult scurvy. J Am Acad Dermatol.

[13] Leggett J, Convery R (2001). Scurvy. N Engl J Med.

[14] Blanchard MS, Romero JM, Hoang MP (2014). Case records of the Massachusetts General Hospital. case 1-2014. a 32-year-old man with loss of vision and a rash. N Engl J Med.

[15] Pimentel L (2003). Scurvy: historical review and current diagnostic approach. Am J Emerg Med.

[16] Chang CY, Rosenthal DI, Mitchell DM, Handa A, Kattapuram SV, Huang AJ (2016). Imaging findings of metabolic bone disease. Radiographics.

[17] Hafez D, Saint S, Griauzde J, Mody R, Meddings J (2016). A deficient diagnosis. N Engl J Med.

[18] Reuler JB (1985). Adult scurvy. JAMA.

